# Effect of Glycolipids Application Combined with Nitrogen Fertilizer Reduction on Maize Nitrogen Use Efficiency and Yield

**DOI:** 10.3390/plants13091222

**Published:** 2024-04-28

**Authors:** Xianghai Meng, Qingshan Dong, Baicheng Wang, Zheng Ni, Xingzhe Zhang, Chunguang Liu, Wenquan Yu, Jie Liu, Xinrui Shi, Dehai Xu, Yan Duan

**Affiliations:** 1 Mudanjiang Branch, Heilongjiang Academy of Agricultural Sciences, Mudanjiang 157000, China; mengxianghai538@163.com (X.M.); mdjdqs@126.com (Q.D.); 13946368993@163.com (B.W.); jxnczxz@163.com (X.Z.); mdjnkybgs@126.com (C.L.); mdjnky@163.com (W.Y.); hour5277@126.com (X.S.); 13946350000@163.com (D.X.); 2 The Centre for Ion Beam Bioengineering Green Agriculture, Hefei Institutes of Physical Science, Chinese Academy of Sciences, Hefei 230031, China; nzlzll@163.com; 3 Heilongjiang Academy of Black Soil Conservation & Utilization, Harbin 150086, China; liujie16777@163.com

**Keywords:** glycolipids, nitrogen reduction, maize yield, net economic benefits, nitrogen fertilizer use efficiency

## Abstract

Microbial-driven N turnover is important in regulating N fertilizer use efficiency through the secretion of metabolites like glycolipids. Currently, our understanding of the potential of glycolipids to partially reduce N fertilizer use and the effects of glycolipids on crop yield and N use efficiency is still limited. Here, a three-year in situ field experiment was conducted with seven treatments: no fertilization (CK); chemical N, phosphorus and potassium (NPK); NPK plus glycolipids (N+PKT); and PK plus glycolipids with 10% (0.9 N+PKT), 20% (0.8 N+PKT), 30% (0.7 N+PKT), and 100% (PKT) N reduction. Compared with NPK, glycolipids with 0–20% N reduction did not significantly reduce maize yields, and also increased N uptake by 6.26–11.07%, but no significant changes in grain or straw N uptake. The N resorption efficiency under 0.9 N+PKT was significantly greater than that under NPK, while the apparent utilization rates of N fertilizer and partial factor productivity of N under 0.9 N+PKT were significantly greater than those under NPK. Although 0.9 N+PKT led to additional labor and input costs, compared with NPK, it had a greater net economic benefit. Our study demonstrates the potential for using glycolipids in agroecosystem management and provides theoretical support for optimizing fertilization strategies.

## 1. Introduction

Chemical nitrogen (N) fertilizer application has received considerable attention due to its irreplaceable functions in regulating soil fertility, crop growth and agriculture sustainability [1,2]. Over the past 50 years, N fertilizer has been extensively utilized as the primary source of reactive N for agricultural production to meet the food demands of a growing population [3,4,5]. To date, it has been reported that global N consumption has exceeded 110 million tons per year, and N consumption is continuing to increase [6]. However, some studies indicate that the plant N fertilizer use efficiency under field conditions is generally less than 35% [7], and abundant N is emitted to the atmosphere or leached into groundwater, resulting in environmental pollution leading to soil acidification, greenhouse effects, groundwater pollution, and other agroecosystem disruptions [8]. Therefore, the systematic application of N fertilizer has become an urgent research topic worldwide.

Soil microorganisms, as important agents of SOM (soil organic matter) formation and decomposition, are pivotal managers of global elemental cycling and balance [9,10]. Microorganisms can contribute to soil N cycling through biological N fixation, regulation of community functions, and the decomposition of exogenous materials and have been extensively studied during the past two decades [11,12]. Recent studies have further revealed that microbial metabolites may regulate plant health and enhance soil fertility [13]. Metabolic exchanges are ubiquitous in natural microbial communities [14]. It is well known that the metabolites of microorganisms are widely used in food processing, drug improvement, cosmetic development, and other fields. In soil, Kost et al. (2023) suggested that metabolic exchanges of microorganisms can alter the dynamic interplay between synergistic and antagonistic interactions, shaping the structure and functions of a given microbial community in soil. Some scholars have shown that the metabolites of actinomycetes can promote crop growth and enhance crop resistance [15]. In addition, Saleem et al. (2019) indicated that microorganisms can secrete nitrogenous compounds in response to soil nutrient stress, which helps to maintain community stability [16]. Thus, soil multifunctionality is achieved, at least in part, through microbial metabolites.

Biological approaches are playing increasingly prominent roles in sustainable agricultural development [17]. Glycolipids are metabolites secreted by soil bacteria belonging to Pseudomonas and are composed of trehalolipid, rhamnolipid, sophorolipod, and mannosylerythritol lipid; these metabolites have been shown to be biodegradable and be acid- and alkali-resistant and have antimicrobial properties with good biocompatibility, which showed that the application of bioglycolipid in soil does not cause severe disturbance to soil animals and microorganisms [18,19,20]. They have a wide range of potential applications in various fields, such as food, pharmaceuticals, environmental remediation, petroleum, and agriculture [21,22,23]. Glycolipids can serve as a cementing substance to promote the formation of macroaggregates and as an energy source to be absorbed by roots to promote crop growth [24]. Additionally, glycolipids have been shown to effectively mitigate greenhouse gas emissions [25]. Notably, the application of glycolipids increased the soil ammonium and nitrate N contents, as well as the glutaminase synthetase activity, in cabbage related to N uptake [26,27]. The use of glycolipids in agricultural production is primarily as biosurfactant. Glycolipids are a type of biosurfactant that has been extensively researched [28]. Compared to chemically synthesized surfactants, biosurfactants (glycolipids) have the advantages of high efficiency and environmental friendliness, in addition to reducing surface tension and stabilizing emulsions [28,29]. Previous studies have demonstrated that glycolipids can be added to foliar fertilizers as a biosurfactant. The hydrophobic groups are adsorbed on the surface of the waxy layer by dispersion force, while hydrophilic groups reach into the fertilizer solution to form a directional adsorption film that replaces the hydrophobic waxy layer [30,31]. This property improves the wetting conditions of foliar fertilizers in the waxy layer and facilitates the use efficiency of fertilizers. However, there is still a lack of understanding regarding the potential of microbial metabolites to substitute for N fertilizer application, improve N fertilizer utilization efficiency, and promote plant growth.

The Northeast Plains of China are the primary areas for grain production in the country, accounting for more than 20% of the annual grain crops [32]. However, the fertility of Mollisols in the Northeast China Plains is decreasing due to frequent cultivation and excessive fertilizer inputs [33]. In this study, a three-year in situ field experiment was conducted to determine the optimal N fertilizer application rate with glycolipids amendment. In this study, we aimed to explain the effects of N fertilizer reduction with glycolipids application on crop yield and N fertilizer use efficiency, as well as the potential of biogglycolipid application in agroecosystems in the Northeast China Plains. Our hypothesis is that the application of glycolipids and a moderate reduction in N fertilizer can enhance crop yields and N fertilizer use efficiency, leading to increased net economic benefits.

## 2. Results

### 2.1. Soil Fertility

**Figure 1:**
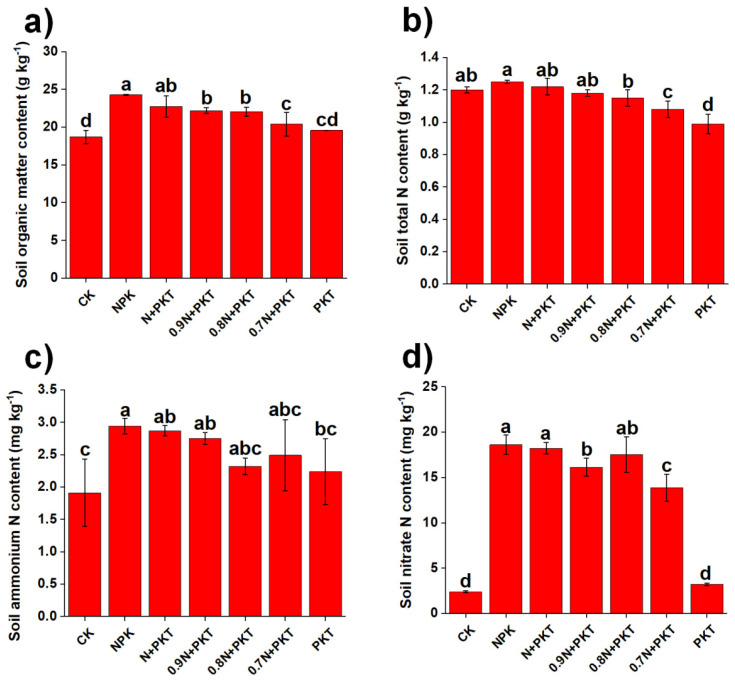
Soil organic matter (**a**), total nitrogen (**b**), ammonium nitrogen (**c**), and nitrate nitrogen (**d**) after 3-year different fertilization. The results show means ± standard deviations (*n* = 3). Different lowercase letters after values indicate a significant difference under different treatments, *p* < 0.05.

Basic soil properties were significantly affected by different fertilization practices (Figure 1 and Table S2). In particular, basic soil properties were significantly affected by different fertilization practices. In terms of the basic soil properties, the different treatments altered N turnover in the soil (Figure 1). The NPK treatment had the highest SOM content, which was significantly greater than that of the other treatments (*p* < 0.05, except for N+PKT). The SOM content under the 0–20% N reduction treatments was greater than that under the 0.7 N+PKT, PKT, and CK treatments (*p* < 0.05). For soil total N, the highest total N content was observed in the NPK treatment, which was significantly greater than that in the 0.8 N+PKT, 0.7 N+PKT, and PKT treatments (*p* < 0.05). Additionally, the total N content under the 0–20% N reduction treatments was greater than that under the 0.7 N+PKT and PKT treatments (*p* < 0.05). The lowest total N content was detected in the PKT treatment group.

The soil N fraction changed after N fertilizer reduction and glycolipids application (Figure 1c,d). Overall, the soil ammonium N and nitrate N contents were higher with glycolipids application and 0–20% N reduction treatments than in CK (*p* < 0.05). Furthermore, N fertilizer input was the key factor in regulating the soil nitrate N content (Figure 1d). The soil nitrate N content was greater in the N addition treatments than in the CK and PKT treatments (*p* < 0.05). Specifically, the soil ammonium N content in the NPK treatment was greater than that in the PKT treatment. Additionally, the soil nitrate N content was greater in the 0–20% N reduction treatment group than in the 0.7 N+PKT treatment group (*p* < 0.05).

The different fertilization practices also affected other soil properties (Table S2). Soil available potassium and pH did not change significantly after 3 years of continuous application of the different fertilization regimes. Soil pH ranged from 7.49 to 7.72, but there was no significant difference between different treatments. Among them, the highest soil pH value was seen in CK, and the lowest was observed in the 0.8N+PKT treatment. However, the soil available phosphorus content was significantly greater in the 0.9 N+PKT treatment than in the CK and PKT treatments (*p* < 0.05). The soil C:N ratio was significantly greater under the N+PKT and 0.9 N+PKT treatments than under the CK treatment (*p* < 0.05) and significantly lower than that under the 0.7 N+PKT treatment (*p* < 0.05). In summary, the use of glycolipids along with a 0–20% reduction in N is a reasonable fertilization strategy for maintaining the fertility of Mollisols.

### 2.2. Plant Growth and Maize Yield

Ear row number, kernel number, ear length, spike-stalk width, and thousand-kernel weight were selected to evaluate the growth of maize under different fertilization regimes (Table S3). The number of ear rows decreased with decreasing N application. Compared with the CK and PKT treatments, the number of ear rows significantly increased in the N+PKT and 0.9 N+PKT treatments (*p* < 0.05). The highest kernel number was found in the 0.7 N + PK treatment, while the lowest was observed in the CN and PKT treatments. N fertilizer is the key factor in increasing kernel number. Compared to the treatments with different levels of N input, the kernel number significantly decreased in the no-N input treatments (CK and PKT) (*p* < 0.05). Compared with those in the CK treatment, the ear length in the NPK, N+PKT, and 0.7 N+PKT treatments significantly increased (*p* < 0.05). The spike-stalk width in the 0.9 N+PKT treatment was greater than that in the CN, 0.7 N+PKT, and PKT treatments (*p* < 0.05). The thousand-kernel weight, which ranged from 269.08 g (CK) to 355.00 g (N+PKT), is a pivotal factor in assessing seed quality and crop yield. Compared to those in the CK and PKT treatments, the thousand-kernel weight significantly increased in the 0–30% N reduction treatments (*p* < 0.05).

**Figure 2:**
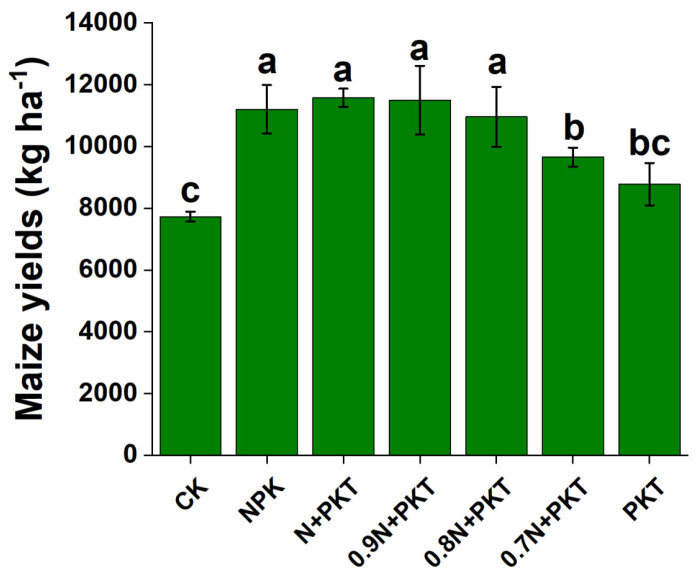
The maize yields after 3-year different fertilization. The results show means ± standard deviations (*n* = 3). Different lowercase letters after values indicate a significant difference under different treatments, *p* < 0.05.

Crop yield is a comprehensive reflection of soil fertility and plant physiological traits under different fertilization regimes and is dependent on N input levels (Figure 2). There were no significant differences in crop yield among the NPK, N+PKT, 0.9 N+PKT, and 0.8 N+PKT treatments; the yields in these treatments were significantly greater than those of the other treatments (*p* < 0.05). Furthermore, the maize yield under the 0.7 N+PKT treatment was significantly greater than that under the CK treatment (*p* < 0.05). In summary, glycolipids can improve soil fertility, promote plant growth, and increase crop yields when N fertilizer application is reduced by 10%. This is the recommended fertilization regime for Mollisols.

### 2.3. N Fertilizer Use Efficiency

**Figure 3:**
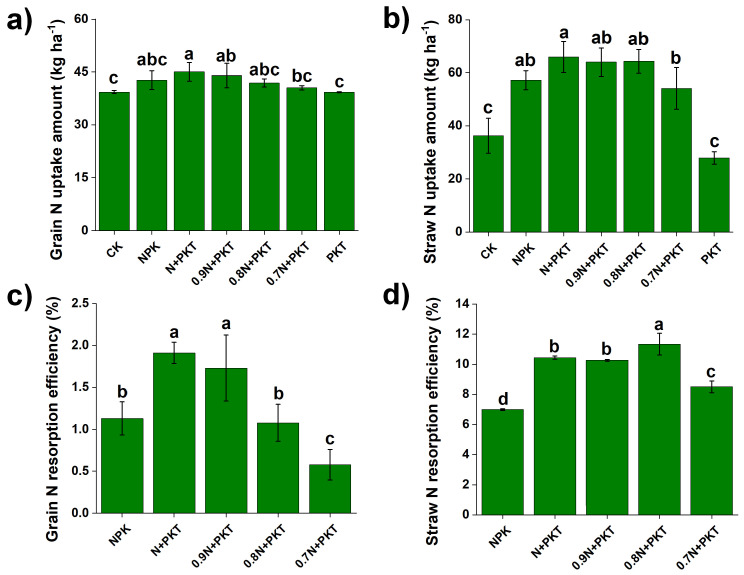
Nitrogen uptake amounts of grain (**a**) and straw (**b**), as well as nitrogen resorption efficiencies of grain (**c**) and straw (**d**) after 3-year different fertilization. The results show means ± standard deviations (*n* = 3). Different lowercase letters after values indicate a significant difference under different treatments, *p* < 0.05.

**Figure 4:**
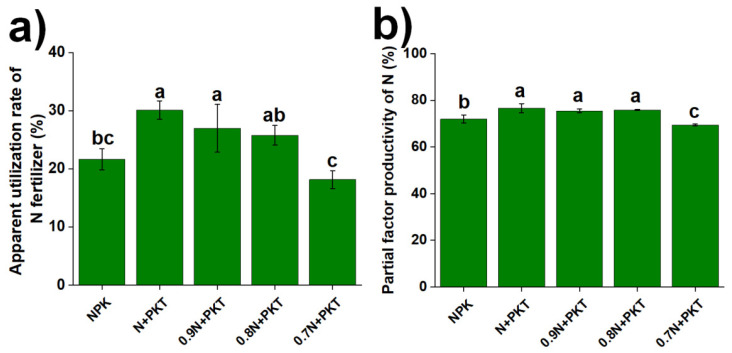
Apparent utilization rate of nitrogen fertilizer (**a**) and partial factor productivity of nitrogen (**b**) after 3-year different fertilization. The results show means ± standard deviations (*n* = 3). Different lowercase letters after values indicate a significant difference under different treatments, *p* < 0.05.

The different fertilization treatments altered the N fertilizer use efficiency of the plants (Figure 3 and Figure 4). Figure 3a,b illustrate the differences in the N uptake amount in grain and straw, respectively. The results indicate that the greatest N uptake in both the grain and straw was observed in the NPK treatment, while the lowest N uptake was observed in the PKT treatment. The N uptake in both the grain and straw under the NPK, N+PKT, 0.9 N+PKT, and 0.8 N+PKT treatments was significantly greater than that under the PKT and CK treatments (*p* < 0.05). Furthermore, changes in the amount of nitrogen taken up resulted in differences in nitrogen recovery efficiency (NRE) (Figure 3c,d). Compared with that in the NPK treatment, the grain NRE in the N+PKT and 0.9 N+PKT treatments significantly increased (*p* < 0.05), while it significantly decreased in the 0.7 N+PKT treatment (*p* < 0.05). The straw NRE was significantly greater in the N+PKT, 0.9 N+PKT, and 0.8 N+PKT treatments than in the NPK and 0.7 N+PKT treatments (*p* < 0.05).

AURN and PFPN are also key indicators of N fertilizer use efficiency and were influenced by the different fertilization regimes (Figure 4). As shown in Figure 4a, AURN ranged from 18.17% for the 0.7 N+PKT treatment to 30.09% for the N+PKT treatment. AURN was significantly greater in the N+PKT and 0.9 N+PKT treatments than in the NPK and 0.7 N+PKT treatments (*p* < 0.05). There was no significant difference in AURN between the N+PKT and 0.9 N+PKT treatments. As shown in Figure 4b, PFPN ranged from 69.47% for the 0.7 N+PKT treatment to 76.72% for the N+PKT treatment. The PFPN was significantly greater under the N+PKT, 0.9 N+PKT, and 0.8 N+PKT treatments than under the NPK and 0.7 N+PKT treatments (*p* < 0.05). In addition, compared with that in the NPK treatment, the PFPN in the 0.7 N+PKT treatment significantly decreased (*p* < 0.05). Therefore, the use of glycolipids along with a 0–20% reduction in N may increase the N fertilizer use efficiency of plants.

### 2.4. The Links between N Fertilizer Input, Soil Basic Property, and N Fertilizer Use Efficiency

**Figure 5:**
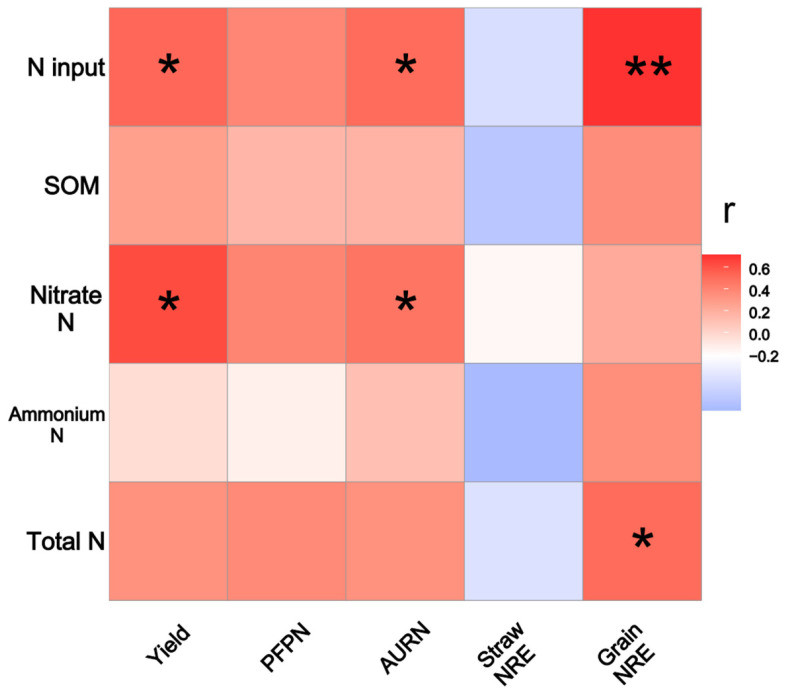
The heatmap indicated the correlations between N fertilizer input level, soil basic properties and N fertilizer use efficiency. The stars * and ** indicate significance at *p* < 0.05, and 0.01, respectively.

Figure 5 shows the close associations between N fertilizer input, basic soil properties, and N fertilizer use efficiency. The results indicated that the N fertilizer input level was positively correlated with crop yield (*p* < 0.05), AURN (*p* < 0.05), and grain NRE (*p* < 0.01). Moreover, the nitrate N content was positively correlated with crop yield (*p* < 0.05) and AURN (*p* < 0.05). Moreover, the total N content and grain NRE were significantly positively correlated (*p* < 0.05).

### 2.5. Net Economic Benefit

As shown in Table 1, among all the calculated specific economic returns (mainly maize output value) and expenses (fertilizer and management in-field costs), these values dominated the total net economic value, and glycolipids application along with a 0–20% N fertilizer reduction provided an additional 1584.02–252.24 ¥/ha/yr of N to the traditional fertilization regime (NPK). Compared with the NPK treatment, the N + PK, 0.9 N + PK, and 0.8 N + PK treatments improved the NEB by 8.28% (1584.02 ¥/ha/yr), 7.80% (1492.32 ¥/ha/yr), and 1.32% (252.34 ¥/ha/yr), respectively. However, 0.7 N+PKT decreased the NEB by 4.75% (908.21 ¥/ha/yr). Furthermore, compared with the NPK treatment, the lowest NEB was observed under the PKT and CK treatments, reaching values of −25.52% (−4882.38 ¥/ha/yr) and −27.22% (−5207.81 ¥/ha/yr), respectively. Hence, the 0.9 N+PKT treatment offered a win–win strategy for reducing N fertilizer application and increasing farmer profits.

## 3. Discussion

### 3.1. Responses of Soil Fertility and Plant Growth to the Addition of N Fertilizer and Glycolipids

In addition to factors such as sunlight, temperature, precipitation, soil texture, and geographical location, appropriate fertilization is one of the most important factors for determining the physiological traits and yield of plants [34]. Fertilizer regimes should be carefully managed to avoid negative impacts on the environment. Fertilization provides essential C sources and nutrients such as C, N, P, and K for crop growth. Furthermore, it enhances soil fertility, mediates greenhouse gas emissions, and increases crop yields [8]. Appropriate fertilization measures are crucial for achieving sustainable agricultural development [35]. SOM is the key to soil fertility [34]. This study revealed that both conventional fertilization and glycolipids application combined with a 0–20% N fertilizer reduction could result in the maintenance of a high SOM content. Generally, exogenous organic material input is a prerequisite for SOM accumulation [36]. According to a Duan et al. (2021) study, N fertilizer input is an important factor in SOM accumulation in the Northeast Plains [13]. Regular N fertilizer input promoted the soil microbial capacity to degrade straw and thus accelerated SOM formation. However, a 50% lower N fertilizer input weakens the ability of microbes to degrade straw, leading to SOM loss. This result pointed out that compared with 50% N fertilizer reduction, more N input was beneficial to stimulate microbial function and promoted soil fertility. And in the present study, glycolipids addition may mitigate the effects of N fertilizer reduction on SOM content [13]. The results indicate that under the premise of adding glycolipids, appropriate reduction of N fertilizer input had no significant effect on SOM content. A previous study also suggested that glycolipids, which act as organic cementing substances, could promote the formation of macroaggregates, which provide physical protection for SOM and promote SOM accumulation [24].

Soil total and mineral N content were identified as key indicators of N supply capacity following a reduction in N fertilization [37,38,39]. The results indicated that compared with conventional fertilization, glycolipids application with a 0–20% nitrogen reduction can allow for the maintenance of the soil N content. The increase in soil total and mineral N is dependent on the amount of N fertilizer applied. However, reducing the amount of N fertilizer can stimulate the functions of N-fixing microorganisms and promote N-fixing efficiency [40]. Wang et al. (2012) demonstrated that the inclusion of rhamnolipid, the main component of glycolipids, can facilitate the transformation of soil organic N into ammonia N [27]. Additionally, Gong et al. (2017) reported that the addition of rhamnolipid can increase the population of cellulose-degrading and nitrogen-fixing microorganisms, leading to an increase in soil N accumulation [41].

Adequate nutrient supply capacity is a prerequisite for crop growth [42]. In this study, the number of ear rows, kernels, ear length, spike-stalk width and thousand-kernel weight were used to assess crop growth performance. The results showed that the 0.9 N+PKT treatment improved maize physiological traits, indicating that crop growth requirements were still met with a 10% reduction in N. Previous studies have shown that rhamnolipid (the main component of glycolipids) can promote crop yields by promoting crop root growth and facilitating rapid plant access to soil nutrients [43]. In addition, the soil C:N ratio is an important indicator of soil health [44]. In this study, the C:N ratio was significantly greater under the N+PKT and 0.9 N+PKT treatments than under the CK treatment and lower than that under the 0.7 N+PKT treatment. This suggests that the C:N ratio needs to be within a reasonable range, which is consistent with previous research [44]. A reasonable C:N ratio has been reported to stimulate microbial functions, promote straw degradation, prevent pathogen infection, and enhance soil fertility, ultimately contributing to greater crop yields [45,46,47,48]. Therefore, 0.9 N+PKT is a beneficial fertilization measure for the sustainable development of agriculture on the Northeast China Plains and for the improvement of farmer benefits.

### 3.2. The Effect of Glycolipids Addition and N Fertilizer Reduction on the N Fertilizer Use Efficiency of Plants

The combination of glycolipids application and N fertilizer reduction not only compensated for the negative effects of N fertilizer deficiencies on soil fertility, crop growth, and yield but also improved N fertilizer efficiency in plants. This study revealed that the N+PKT and 0.9 N+PKT treatments significantly increased the maize NRE, AURN, and PFPN compared to those under no fertilization. Our results suggest the potential functions of glycolipids in promoting the N fertilizer use efficiency of plants. Previous studies have shown that the addition of glycolipids increases the uptake of essential plant micronutrients, such as Fe^2+^, Mn^2+^, Cu^2+^, and Zn^2+^, in soybeans, which promotes crop growth and yield [49]. In addition, studies have shown that the application of glycolipids can increase soil N-cycling enzyme activity and increase plant free amino acid content, which implies that more mineral N is stored in the soil to promote crop growth [50]. Furthermore, the use of glycolipids has been shown to increase plant root biomass, thereby enhancing the ability of crops to obtain N from the soil [46]. 

Glycolipids can form chelates with some of the ions in the soil [51]. By taking advantage of the fact that glycolipids can easily enter the cells, they can transport other substances, such as nutrients, and make them more easily utilized by plant cells [52]. In addition, glycolipids themselves can also act as a nutrients source for the plant root, thus promoting plants growth [29]. Moreover, as biosurfactant, glycolipids can improve the activity of β-glucosidase and cellulase, promote the degradation of straw, and provide energy and nutrition for crops [31]. Therefore, glycolipids also have great potential in soil fertility improvement.

N fertilizer input is crucial for regulating N use efficiency in plants. The results of this study revealed close associations of the N fertilization rate and nitrate N content with N fertilizer use efficiency (Figure 5). Nitrate N rather than ammonium N is the predominant form of N present in dryland soils [53]. A recent study indicated that nitrate signaling promotes plant growth by upregulating gibberellin biosynthesis and destabilizing DELLA proteins [54], which explained the internal mechanism by which nitrate N promotes plant growth; our findings also verified these results (Figure 5). Generally, glycolipids affect plant N fertilizer utilization in two ways: (i) by improving soil N-cycling enzyme activity to provide more available N for crop growth and (ii) by promoting root growth to increase nutrient absorption capacity. In addition, the application of 0.9 N+PKT has been found to increase net economic benefits by reducing fertilizer expenses and increasing crop yields. 

Under the premise of maintaining soil fertility, crop growth, and yield, the NEB of farmers is an important factor to consider for modified fertilization strategies. In this study, based on local prices, the 0.9 N+PKT treatment increased expenses associated with glycolipids input by 37.5 ¥/ha/yr but reduced expenses for nitrogen fertilizer input by 82.5 ¥/ha/yr. As a result, the income from maize increased by 1447.32 ¥/ha/yr compared to that from conventional fertilization, resulting in an NEB of 1492.32 ¥/ha/yr. The addition of glycolipids can be a reliable strategy for maintaining soil fertility and productivity in Mollisols on the Northeast China Plains, with a 10% reduction in N fertilizer input.

## 4. Materials and Methods

### 4.1. Site Description and Sampling 

A field experiment was established in 2021 in Wenchun (44°59′61″ N, 129°59′18″ E), Mudanjiang city, Heilongjiang Province, Northeast China Plains, which is an important grain-producing area. This region has a typical temperate continental monsoon climate with an average annual temperature of 5.0 °C and a mean annual precipitation of 579.7 mm. The soil is classified as a meadow soil according to US Soil Taxonomy (USST). The cropping system consisted of a continuous maize (*Zea mays* L.) monoculture.

The following seven treatments were applied for 3 years: (1) no fertilization (CK); (2) regular chemical fertilization (NPK, 84.00 kg N/ha, 40.17 kg P/ha, 62.23 kg K/ha); (3) regular chemical fertilization with glycolipids (N+PKT, 84 kg N/ha, 40.17 kg P/ha, 62.23 kg K/ha, 0.75 kg/ha of glycolipids); (4) regular P, K, and 10% N reduction with glycolipids (0.9 N+PKT, 75.60 kg N/ha, 40.17 kg P/ha, 62.23 kg K/ha with 0.75 kg/ha of glycolipids); and (5) regular P, K, and 20% N reduction with glycolipids (0.8 N+PKT, 67.20 kg N/ha, 40.17 kg P/ha, 62.23 kg K/ha with 0.75 kg/ha of glycolipids); (6) regular P, K, and 30% N reduction with glycolipids (0.7 N+PKT, 58.80 kg N/ha, 40.17 kg P/ha, 62.23 kg K/ha with 0.75 kg/ha of glycolipids); (7) regular P, K, with glycolipids (PKT, 40.17 kg P/ha, 62.23 kg K/ha with 0.75 kg/ha of glycolipids). Generally, N was applied by urea (N 16%) and diammonium phosphate (N 18%); P and K were applied by diammonium phosphate (P_2_O_5_ 46%) and potassium chloride (K_2_O 60%). Glycolipids are the compounds products that are composed of rhamnolipid (main component, account for about 70%), trehalolipid, sophorolipod, and mannosylerythritol lipid (account for about 30%). All of these components were collected through microbial secretion (such as Pseudomonasaeruginosa). Glycolipids are freeze-dried to form a white powder product, which is dissolved in water and evenly sprayed on the soil. In this study, glycolipids (0.75 kg/ha) were applied to evaluate the potential of N fertilizer reduction, plant growth, and yield. It was noted that straw return was carried out in all the above treatments, and all other management practices were consistent among the treatments during the experiment.

Soils were sampled after the maize harvest in October 2023. A completely randomized block design consisting of 7 treatments with 3 replicates was adopted in this study. Each field plot was 0.65 m × 9 m. We collected 9 soil cores (5 cm diameter) from the top 20 cm of bulk soil in each plot. Each soil sample consisted of a mixture of subsamples randomly collected from 9 different positions in the same plot. In total, 21 soil samples were collected from 7 treatments with 3 replicates. The soils were sieved through a 2 mm mesh, mineral particles was carefully removed, and then the soils were homogenized and stored in an incubator at 4 °C in a 40% moisture environment.

The plants were sampled during the maize harvest in October 2023. We randomly collected 3 plants (including straw and grain) as replicates from each field plot, and 3 replicates were collected for each treatment. In total, 21 straw/biomass samples were collected from 7 treatments with 3 replicates. Subsequently, the straw and grain of the plants were carefully separated for further analysis.

### 4.2. Soil Basic Chemical Properties and Plant Physiological Traits

Soil pH was measured at a soil/water ratio of 1:2.5 (weight/weight). Air-dried soil and 25 mL of deionized water were shaken together for 1 min, settling was allowed to occur for 30 min, and the soil pH was determined using an electrode. Soil organic carbon (SOC) was measured by titrimetry after soil oxidation with a mixture of H_2_SO_4_ and K_2_Cr_2_O_7_. Total N was determined using the Kjeldahl method; total and available P were determined using molybdenum blue colorimetric methods; total K and available K were determined using flame photometry; and SOM was determined by titrimetry after soil oxidation with a mixture of H_2_SO_4_ and K_2_Cr_2_O_7_. Soil ammonium and nitrate N were determined by a Kelvin nitrogen determination instrument. All of these methods have been described in Lu (2000) [55]. The basic soil chemical properties before the different fertilization treatments were applied are shown in Table S1.

The number of ear rows and kernels was measured by visual counting; ear length and spike-stalk width were measured by a graduated ruler; and thousand-kernel weight was measured by a quantitative counting plate and centesimal balance. The total N of straw and grain was determined using the Kjeldahl method.

### 4.3. N Fertility Use Efficiency Evaluation and Calculation of Net Economic Benefits

N resorption efficiency (NRE) [56], apparent utilization rate of N fertilizer (AURN), [57] and partial factor productivity of N (PFPN) [58] were calculated to evaluate the N fertility use efficiency of plant.

NRE (%) = (N_applied_ − N_no-apply_)/N_input_ × 100; (1)

where N_applied_ and N_applied_ are the N uptake amount under N application conditions (kg) and the N uptake amount without N application (kg), respectively. N_input_ is the N fertilizer input amount (kg).

AURN (%) = N_applied_/N_input_ × 100; (2)

where N_applied_ and N_input_ are the N uptake amount under N application conditions (kg) and the N fertilizer input amount (kg), respectively.

PNPF (kg/kg) = Crop yield_applied_/N_input_; (3)

where Crop yield_applied_ and N_input_ are crop yields under N application conditions (kg) and N fertilizer input amounts (kg), respectively.

The net economic benefit (NEB) was calculated to assess the utilization potential of glycolipids.

NEB (¥/ha/yr) = V_income_ − V_expenses_ (4)

where V_income_ is the maize output value and V_expenses_ includes the expenses of fertilizer and management cost in fields.

### 4.4. Statistical Analysis

The soil and plant properties were subjected to the chi-squared test for independence of variance analysis. Significant differences were determined by one-way analysis of variance (ANOVA) based on the post hoc Tukey test at the 5% level. Prior to ANOVA, normality and homogeneity of variances were tested by the Kolmogorov–Smirnov test and Levene’s test, respectively. If the normality condition was not met, log or square-root transformation was performed. One-way ANOVA was carried out in SPSS 21.0 (SPSS Inc., Chicago, IL, USA). The heatmap was performed using the function “heatmap.2” in the R package “ggplots”.

## 5. Conclusions

After 3 consecutive years of glycolipids application and reduced N fertilization, we observed changes in soil fertility, crop physiological traits, yield, and N fertilizer use efficiency. The 0.8 N+PKT treatment was found to maintain soil fertility (SOM and N fraction), while the 0.9 N+PKT treatment promoted plant growth and crop yields compared to the NPK treatment. In addition, the N fertilizer input level and nitrate N content were the main factors regulating the N fertilizer use efficiency of plants. Compared with the NPK treatment, the 0.9 N+PKT and 0.8 N+PKT treatments increased the NRE, AURN, and PFPN of plants by promoting plant root growth and maintaining adequate soil labile N content. It is also worth noting that the application of glycolipids along with a 10% N fertilizer reduction resulted in a high NEB for local farmers. Therefore, the 0.9 N+PKT treatment not only improved soil fertility, plant physiological traits, and crop yield but also efficiently reduced excessive N fertilizer use, improved N fertilizer use efficiency, provided considerable economic benefits for farmers, and is a win–win strategy in the Northeast China Plains.

## Figures and Tables

**Table 1:** The economic value of income and expenses after 3-year different fertilization.

Treatment	Maize Yields (kg/ha)	Maize Output Value (¥/ha/yr)	Fertilizer Cost (¥/ha/yr)	Management Cost (¥/ha/yr)	Net Income (¥/ha/yr)	Rate of Change Compared with NPK Treatment (%)
CK	7727.35	17,772.91	0.00	3850.00	13,922.91	−27.22
NPK	11,205.38	25,005.72	2025.00	3850.00	19,130.72	——
N+PKT	11,577.06	26,627.24	2062.50	3850.00	20,714.74	8.28
0.9 N+PKT	11,501.32	26,453.04	1980.00	3850.00	20,623.04	7.80
0.8 N+PKT	10,960.01	25,208.02	1974.96	3850.00	19,383.06	1.32
0.7 N+PKT	9656.26	24,126.06	2053.54	3850.00	18,222.51	−4.75
PKT	8312.32	19,118.34	1020.00	3850.00	14,248.34	−25.52

## Data Availability

The raw data that support the findings of this study are available on request from the authors.

## Supplementary Materials

The following supporting information can be downloaded at: https://www.mdpi.com/article/10.3390/plants13091222/s1, Table S1: The initial and treated basic soil chemical properties under different fertilization treatments during experiment periods. Table S2: Soil basic properties after 3-year different fertilization. Table S3: Maize physiological traits after 3-year different fertilization.
