# Supplementary material for: Effect of Glycolipids Application Combined with Nitrogen Fertilizer Reduction on Maize Nitrogen Use Efficiency and Yield
Source: Plants (Basel). 2024 Apr 28;13(9):1222. doi: 10.3390/plants13091222 (PMC11085625; doi:10.3390/plants13091222)
Supplement: Supplementary file 1 [file plants-13-01222-s001.zip › plants-2964016-supplementary.pdf]

Table S1 The initial and treated basic soil chemical properties under different fertilization treatments during experiment periods

Time	Treatments	pH	Soil organic matter (g kg^{-1})	Soil total nitrogen (g kg^{-1})	Soil total phosphorus (g kg^{-1})	Soil total potassium (g kg^{-1})	Soil available phosphorus (mg kg^{-1})	Soil available potassium (mg kg^{-1})
**2020**	**Initial soil**	7.90±0.31	22.14±3.70	1.18±0.29	0.71±0.10	22.53±5.57	51.52±7.02	179.18±30.41
	**CK**	7.88±0.44	20.48±2.97	1.10±0.31	/	/	48.77±11.52	184.04±25.47
	**NPK**	7.48±0.61	23.41±2.96	1.21±0.24	/	/	53.87±6.27	171.84±40.26
	**N+PKT**	7.51±0.34	22.48±4.10	1.23±0.18	/	/	52.77±5.79	188.84±26.64
**2021**	**0.9N+PKT**	7.52±0.87	22.17±0.94	1.19±0.21	/	/	59.18±10.69	179.29±30.58
	**0.8N+PKT**	7.69±0.48	22.27±2.87	1.20±0.22	/	/	58.48±9.57	169.94±29.37
	**0.7N+PKT**	7.76±0.41	21.09±3.97	1.14±0.36	/	/	54.57±8.47	173.29±32.71
	**PKT**	7.71±0.51	21.67±3.88	1.08±0.46	/	/	50.66±14.98	161.08±17.65
	**CK**	7.79±0.70	19.01±5.47	1.12±0.24	/	/	48.91±6.78	163.81±18.95
	**NPK**	7.53±0.15	23.88±2.17	1.22±0.15	/	/	56.87±7.69	182.51±40.20
	**N+PKT**	7.57±0.42	23.08±3.14	1.21±0.24	/	/	55.57±10.28	179.26±30.48
**2022**	**0.9N+PKT**	7.56±0.26	23.91±1.69	1.23±0.24	/	/	57.33±4.90	173.26±16.54
	**0.8N+PKT**	7.66±0.18	23.42±2.75	1.14±0.11	/	/	57.61±10.22	177.40±19.64
	**0.7N+PKT**	7.66±0.20	21.68±2.00	1.09±0.21	/	/	53.21±1.97	170.25±8.23
	**PKT**	7.71±0.26	21.00±3.41	1.09±0.34	/	/	55.58±8.21	155.48±17.09

The results show means ± standard deviations (n = 3).

Table S2 Soil basic properties after 3-year different fertilization

Treatment	pH	Soil C:N ratio	Soil available phosphorus (mg kg^{-1})	Soil available potassium (mg kg^{-1})
**CK**	7.72±0.069 a	9.01±0.46 b	46.74±1.23 b	155.49±9.53 a
**NPK**	7.54±0.087 a	11.29±0.051 a	57.92±5.19 ab	178.00±32.97 a
**N+PKT**	7.59±0.026 a	10.80±0.21 a	58.83±10.03 ab	172.49±5.77 a
**0.9N+PKT**	7.5±0.050 a	10.87±0.28 a	61.12±13.92 a	177.34±27.91 a
**0.8N+PKT**	7.49±0.036 a	11.10±0.24 a	58.62±6.70 ab	172.56±12.53 a
**0.7N+PKT**	7.58±0.020 a	10.97±0.25 a	59.94±1.47 ab	165.46±9.05 a
**PKT**	7.51±0.026 a	11.86±0.58 a	46.82±1.26 b	145.30±3.59 a

The results show means ± standard deviations (n = 3). Different lowercase letters after values indicate a significant difference under different treatments, $P < 0.05$.

Table S3 Maize physiological traits after 3-year different fertilization

Treatment	Ear rows number	Kernel number	Ear length (cm)	Spike-stalk width (cm)	Thousand kernel weight (g)
**CK**	14.67±0.58 bc	38.33±0.58 d	19.35±0.87 c	46.17±0.04 e	269.08±3.35 e
**NPK**	15.67±0.58 ab	42.33±0.58 ab	21.32±0.75 ab	50.48±0.06 ab	344.87±10.66 b
**N+PKT**	16.33±0.58 a	42.00±1.00 ab	21.10±1.25 ab	49.54±0.07 c	355.00±0.09 a
**0.9N+PKT**	16.33±0.58 a	41.33±0.58 bc	20.79±0.37 abc	50.79±0.10 a	337.82±4.02 bc
**0.8N+PKT**	15.33±0.58 abc	40.67±0.58 c	20.49±0.04 bc	49.95±0.08 bc	338.25±2.67 bc
**0.7N+PKT**	15.33±0.58 abc	42.67±0.58 a	22.11±0.12 a	48.67±0.85 d	330.83±2.67 c
**PKT**	14.33±0.58 c	38.33±0.58 d	20.11±1.25 bc	48.56±0.60 d	285.41±5.61 d

The results show means ± standard deviations (n = 3). Different lowercase letters after values indicate a significant difference under different treatments, $P < 0.05$.
